# Synthesis of Hierarchical Porous SAPO-34 and Its Catalytic Activity for 4,6-Dimethyldibenzothiophene

**DOI:** 10.3389/fchem.2022.854664

**Published:** 2022-03-14

**Authors:** Hua-Qin Wang, Yun-Qi Cui, Ya-Long Ding, Mei Xiang, Pei Yu, Rong-Qiang Li

**Affiliations:** ^1^ College of Chemistry and Pharmaceutical Engineering, Huanghuai University, Zhumadian, China; ^2^ School of Chemical Engineering and Materials, Changzhou Institute of Technology, Changzhou, China

**Keywords:** hierarchical, zeolite, SAPO-34, material and process optimization, 4,6-dimethyldibenzothiophene (4,6-DMDBT)

## Abstract

Zeolite SAPO-34 has been widely used in the industry because of its special pore structure and wide distribution of acid sites in the pore channel. However, traditional SAPO-34 with a small pore size suffers from carbon deposition and deactivation in catalytic reactions, and its inability to catalytically convert bulky organic molecules limits its industrial application. Meanwhile, impurities of SAPO-5, which have weak acidity leading to rapid catalyst deactivation, appear in SAPO-34 zeolite. Therefore, it is of great significance to synthesize SAPO-34 zeolite with a mesoporous pore structure, which can significantly improve the transfer of molecules in zeolites. In this paper, SAPO-34 zeolite with a hierarchical pore structure was synthesized, and its hydrodesulfurization performance for 4,6-dimethyldibenzothiophene (4,6-DMDBT) was studied in a fixed bed reactor. The characteristic results show that BET-specific surface area, micropore volume, and mesoporous volume of synthesized SAPO-34 are 754 m^2^ g^−1^, 0.25, and 0.23 cm^3^ g^−1^ respectively, and the pore size is mainly concentrated at 4 nm. The catalytic conversion of 4,6-DMDMT with Co- and Mo-supported SAPO-34 is about 83%, which is much higher than the catalytic performance of Al_2_O_3_.

## Introduction

Aluminophosphate (AlPO) zeolite is composed of alternating AlO_4_ and PO_4_ tetrahedrons (Wilson et al., 1982), and has no Bronsted acidity due to the absence of exchangeable electric charge in its framework. Compared with the silicoaluminite zeolite, AlPO zeolite has rich diverse varieties and strong adjustability (Rakoczy et al., 1999), but the small pore size limits its application in the catalytic conversion of bulky organic molecules (Carreon et al., 2008). In the synthesis of silica alumina zeolites, the introduction of a mesoporous structure using surfactant as a structure directing agent (SDA) makes it possible to synthesize mesoporous AlPO zeolite. In 1993, Czametzki first synthesized mesoporous AlPO zeolite with weak catalytic ability because of charge neutrality or weak acidity of the zeolite framework (Kranshaar-Czametzki et al., 1993). The type of mesoporous aluminophosphate zeolite varies with synthesis conditions. The formation of layered mesoporous AlPO_4_ is more favorable at higher crystallization temperature and the initial solution containing SiO_4_. Tiemann et al. firstly reported the synthesis of mesoporous layered aluminum phosphate material SCS-22, but the mesoporous layered structure collapsed easily during material roasting process (Tiemann and Fröba, 2001). Afterwards, Fu et al. synthesized layered mesoporous aluminum phosphate material by using long-chain alkylammonium bromide [C_n_H_2n+l_ (CH_3_)_3_)NBr] (Fu et al., 1995). Kimura et al. found that pure layered and hexagonal mesoporous zeolite can be synthesized by using hexadecyl ammonium chloride as SDA (Kimura et al., 1999).

As for the absence of acidity resulting from lack of exchangeable electric charge in framework, doping other atoms into the framework of aluminophosphate zeolites is one of the effective methods to improve the catalytic activity of zeolites. Zhao et al. firstly used the synthesis method of mesoporous aluminophosphate zeolite (AlPO) in a mesoporous silicoaluminophosphate zeolite field and found that the strong ion pairing effect between sodium ions and aluminum phosphate species was harmful to the formation of a mesoporous structure (Zhao et al., 1997; Luan et al., 1998). Compared with mesoporous silicon materials, mesoporous phosphorous aluminum materials started late, and there are few reports on their applications, but their excellent catalytic activity and the easy introduction of metal atoms have attracted the special attention of researchers. For example, mesoporous Fe-AlPO can efficiently catalyze the oxidation of cyclohexane under neutral conditions, and the catalyst has a long service life. Mesoporous Co-AlPO has high selectivity for the hydrogenation of nitro and carbonyl compounds. Cr-AlPO and Ti-AlPO zeolites have high catalytic activity for the oxidation of cyclohexane and the alkyl substitution of carbonyl compounds under neutral conditions, respectively.

With the introduction of Si atoms into the framework of aluminophosphate zeolites where Si atoms can enter the framework of zeolites by substituting Al or P atoms, named SAPO zeolites (Lok et al., 1984; Chen et al., 1999; Arstad and Kolboe, 2001), the acidity and catalytic performance have been greatly improved as a result of the presence of exchangeable electric charges, though the thermal stability of the zeolites decreased (Numpilai et al., 2021). Like mesoporous aluminosilicate zeolites, mesoporous SAPO zeolites have potential applications in the fields of catalyst, drug delivery, and gas and liquid adsorption, and have important application value in the fields of chemical industry, biotechnology, and environmental energy, among others (Song et al., 2001; Hwang et al., 2016). SAPO-34 is a member of a series of SAPO zeolites, where eight-membered rings in the crystal are linked into an ellipsoidal cavity. The crystal structure of SAPO-34 is similar to that of chabazite, belonging to the CHA structure, and the regularly arranged cages form three-dimensional channels with 8-ring openings of 0.34 nm (Chen et al., 2007; Tian et al., 2015; Jiang et al., 2021; Lin et al., 2021). SAPO-34 crystal has high hydrothermal stability, and its special eight-membered ring pore structure can effectively inhibit the formation of aromatics, and has a high selectivity for light olefins, up to 90% (Hwang et al., 2019).

Traditional microporous SAPO-34 is favored by researchers because of its high catalytic activity, high selectivity, and good hydrothermal stability in MTO reaction (Liang, et al., 2021). However, traditional SAPO-34 has a small pore size and is prone to carbon deposition and deactivation in catalytic reaction of bulky organic molecules, which cannot meet the needs of the development of the chemical industry (Zhong et al., 2017; Kim et al., 2021; Zhang et al., 2021). To cope with these shortcomings, several strategies have been developed to improve the diffusion limitation of SAPO-34, such as synthesis of nano-sized mesopore-containing SAPO-34 (Yang et al., 2014; Sun et al., 2018; Mi et al., 2021). Hierarchical porous SAPO-34 can be synthesized by post-treatment, such as acid or alkali treatment, in which the skeleton cations were removed selectively. Besides, the hierarchical porous SAPO-34 can also be synthesized by using a hard template method, which often refers to the application of mesoporous carbon particles or carbon nanotubes, and a soft template method, which refers to the use of organo-silane, surfactants, and polymers (Schmidt et al., 2012; Rimaz et al., 2016; Varzaneh et al., 2016). Although some researchers reported the synthesis of mesoporous SAPO-34 (Li et al., 2014; Shi et al., 2021), the expensive long-chain amines used in the synthesis method seriously hindered its industrial application (Sun et al., 2021; Xuan et al., 2021). In this paper, hierarchical porous SAPO-34 was synthesized by using diethylamine (DEA) and morpholine as SDA, silicone quaternary ammonium salt {[(C_2_H_5_O)_3_SiC_3_H_6_N^+^(CH_3_)_2_C_18_H_37_]Br} as mesoporous SDA, and phosphoric acid, pseudo boehmite, and silica sol as phosphorus source, aluminum source, and silicon source, respectively. The effects of the amount of DEA and morpholine, phosphoric acid, silica sol, and silicone quaternary ammonium salt on the synthetic products were investigated. The hydrodesulfurization of 4,6-dimethyl dibenzothiophene (4,6-DMDBT), which is difficult to remove from diesel fraction, was studied in a fixed bed reactor with hierarchical porous SAPO-34-supported Co and Mo metal sulfide as catalysts.

## Experimental Section

### Synthesis of Microporous SAPO-34

#### Synthesis of Microporous SAPO-34 Using Organic Silicon Source and Organic Aluminum Source

The solution composed of a certain amount of DEA and tetraethyl orthosilicate (TEOS) was added into another solution that contained a certain amount of phosphoric acid (H_3_PO_4_, 85 wt%) and aluminum isopropoxide solution, and the material ratio of the final white mushy mixture was 1.0 Al_2_O_3_:1.0 P_2_O_5_:0.6 SiO_2_:2.0 DEA:50 H_2_O, and then the mixture was transferred into an autoclave lined with polytetrafluoroethylene. After the autoclave was kept at 200°C for 48 h, products obtained were filtered, dried at 120°C for 12 h, and roasted at 550°C for 4 h to remove organic SDA. The obtained white powder was microporous SAPO-34, labeled as SAPO-34-M1.

#### Synthesis of Microporous SAPO-34 Using Inorganic Silicon Source and Inorganic Aluminum Source

Some phosphoric acid (H_3_PO_4_, 85%) was added dropwise into a certain amount of pseudo boehmite solution, and stirred at room temperature for 2 h. Then, some silica sol is added to the system, followed by the addition of a certain amount of DEA. In the process of dropping DEA, the reaction system thickened first, then diluted, and finally showed a white mushy mixture with the same material ratio as 1.0 Al_2_O_3_:1.0 P_2_O_5_:0.6 SiO_2_:2.0 DEA:50 H_2_O. Subsequent treatment is the same as synthesis of microporous SAPO-34 using organic silicon source and organic aluminum source, and the obtained white powder was also microporous SAPO-34, labeled as SAPO-34-M2.

### Synthesis of Hierarchical Porous SAPO-34

Except for the fact that a certain amount of mesoporous SDA {[(C_2_H_5_O)_3_SiC_3_H_6_N^+^(CH_3_)_2_C_18_H_37_]Br} was added into the synthesis system before the addition of DEA, the other synthesis steps are the same as the synthesis of microporous SAPO-34 using inorganic silicon source and inorganic aluminum source. The obtained white powder was hierarchical porous SAPO-34, labeled as HSAPO-34.

### Preparation of Catalysts

The zeolite sample was soaked with solution of ammonia solution of (NH_4_)_2_MoO_4_, Co(NO_3_)_2_, and complexing agent EDTA by equal volume impregnation method. The impregnated sample was kept at room temperature for 12 h, then at 100°C for 12 h, and then pressed and ground to particles whose diameter was between 0.245 and 0.350 mm. Subsequently, the sample was heated to 400°C in H_2_S atmosphere and maintained for 3 h to obtain the corresponding catalyst.

### Characterization and Catalytic Performance of SAPO-34

The x-ray powder diffraction (XRD) of SAPO-34 was carried out on a RIGAKU ultimate V diffractometer, and the ray emission source was Cu Target Kα. The radiation tube voltage and current are 40 kV and 40 mA, respectively. The scanning speed is 2°·min^−1^, and the scanning range is 5–40°. The N_2_ adsorption–desorption isotherm of SAPO-34 was characterized on a micromeritics ASAP2020 analyzer at liquid nitrogen temperature. The sample was pretreated under vacuum at 350°C for 10 h before analysis. The specific surface area of the sample was calculated by the Barrett–Emmett–Teller (BET) method, and the pore size distribution was calculated by the Barrett–Joyner–Halenda (BJH) method. The scanning electron microscopy (SEM) image was obtained from JSM-600, and the sample was ground with agate mortar and sprayed with gold before the test.

The catalytic performance of catalyst was conducted on a fixed bed reactor. A small amount of quartz cotton is padded at the bottom of the fixed bed reactor to prevent the catalyst from flowing out of the constant temperature zone of the reactor. The prepared catalyst is mixed with quartz sand with the same size of 0.245–0.350 mm, so that the catalyst can be well dispersed, so as to prevent the catalyst from aggregation and from making contact with the reactant efficiently during the reaction process. After the mixture of catalyst and quartz sand is loaded into the reactor, a small amount of quartz sand and quartz cotton are successively loaded on the top of the reactor, which can stabilize the flow speed of the reaction mixture in the reaction process. 4,6-Dimethyldibenzothiophene was dissolved in decahydronaphthalene to prepare a solution with a mass fraction of 4,6-DMDBT of 0.5 wt% as reaction mixture, and hydrodesulfurization reaction was carried out at 290°C and 5.0 MPa. Meanwhile, γ-Al_2_O_3_ was also used as the catalyst support for the same catalytic reaction.

## Results and Discussion

### Characterization of SAPO-34 Zeolite

#### X-Ray Diffraction

##### Effects of Different Raw Materials

**FIGURE 1:**
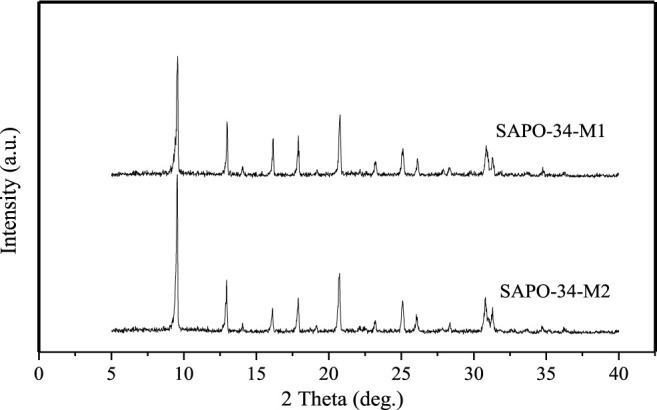
Comparison of XRD pattern of SAPO-34-M1 and SAPO-34-M2.

The main raw materials for the synthesis of SAPO-34 are silicon source, aluminum source, and phosphoric acid. Fu Ye et al. studied SAPO-34 prepared with different silicon and aluminum sources and found that the zeolite synthesized with inorganic silicon and aluminum sources has the highest crystallinity, while the zeolite synthesized with organic silicon and aluminum sources has the lowest crystallinity, and the effect of silicon source on crystallinity is smaller than that of the aluminum source (Fu et al., 2001). Popova et al. further studied the effect of SAPO-34 samples synthesized from different silicon and aluminum sources on MTO reaction, and experimental results showed that the samples synthesized from organic silicon and aluminum sources inactivated faster in the reaction (Popova et al., 1998). In contrast, the samples synthesized from inorganic silicon and aluminum sources had better crystallinity and higher stability. Herein, Figure 1 shows the comparison of XRD patterns of SAPO-34-M1 and SAPO-34-M2 with the same material ratio as 1.0 Al_2_O_3_:1.0 P_2_O_5_:0.6 SiO_2_:2.0 DEA:50 H_2_O, which were synthesized from organic silicon and aluminum sources (TEOS and aluminum isopropoxide) and inorganic silicon and aluminum sources (silica sol and pseudo boehmite), respectively. SAPO-34-M2 is taken as the reference and its crystallinity is assumed to be 100%; the relative crystallinity of SAPO-34-M1 is calculated to be 95%. This result shows that SAPO-34-M2 synthesized from an inorganic silicon source (silica sol) and an inorganic aluminum source (pseudo boehmite) also has high crystallinity.

##### Influence of Material Ratio

**FIGURE 2:**
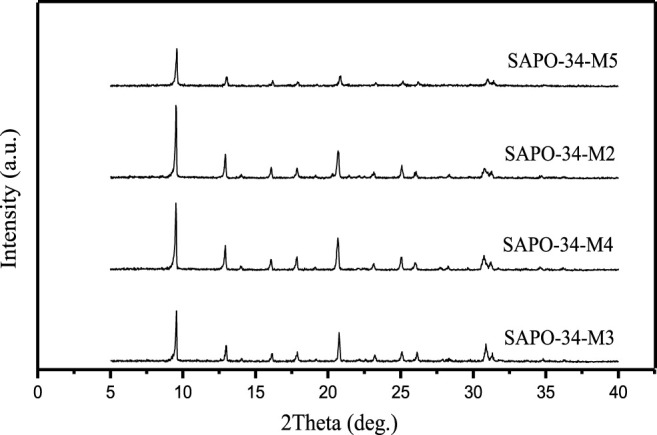
Different XRD patterns of SAPO-34 zeolites synthesized with different silico content when inorganic silicon source (silica sol) and inorganic aluminum source (pseudo boehmite) are used.

In the synthesis of SAPO-34, the material ratio has a great influence on the type of molecular sieve of the synthetic product. Li et al. pointed out that when the molar ratio of phosphorus to aluminum in the synthetic system is greater than 1, the synthetic product is mainly phosphoaluminate; when this molar ratio is less than 1, a series of SAPO-34 zeolite can be synthesized (Li et al., 1987). It is also pointed out that when TEAOH/P_2_O_5_ in the system is 2.0–3.0, the synthetic product is pure SAPO-34, and the increase of the amount of TEAOH is beneficial to the synthesis of SAPO-34. Even when the silicon source, aluminum source, phosphorus source, and SDA are the same, different types of SAPO zeolite, such as SAPO-11, SAPO -31, SAPO-34, and SAPO-41, can be synthesized when the material ratio is different. Figure 2 is the XRD pattern of SAPO-34 zeolites synthesized with DEA as SDA when the silicon content in the material was different. The material compositions of each sample were as follows:

1.0 Al_2_O_3_:1.0 P_2_O_5_:0.1 SiO_2_:2.0 DEA:50 H_2_O (SAPO-34-M3).

1.0 Al_2_O_3_:1.0 P_2_O_5_:0.3 SiO_2_:2.0 DEA:50 H_2_O (SAPO-34-M4).

1.0 Al_2_O_3_:1.0 P_2_O_5_:0.6 SiO_2_:2.0 DEA:50 H_2_O (SAPO-34-M2).

1.0 Al_2_O_3_:1.0 P_2_O_5_:1.0 SiO_2_:2.0 DEA:50 H_2_O (SAPO-34-M5).

The calculation shows that the crystallinity of the sample is as follows: SAPO-34-M2 (100%) > B (95%) > A (94%) > D (79%). When the silicon content in the material is small (A and B), the crystallinity of the sample is equivalent to C, and when the silicon content in the material increases to a certain extent, the crystallinity (D) of the sample decreases.

##### Effects of Different SDA

**FIGURE 3:**
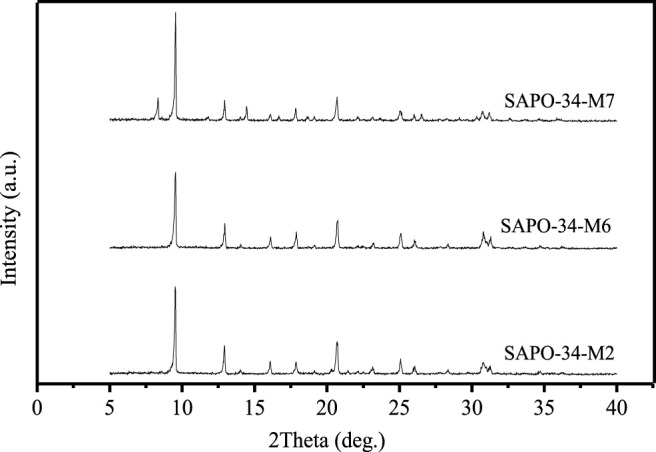
XRD patterns of SAPO-34 samples synthesized with different SDA.

The type and amount of SDA are crucial to the formation of the crystal structure, because SDA not only guides the formation of the crystal structure, but also controls the distribution of silicon atoms on zeolite skeleton to a certain extent. Li et al. used the mixture of triethylamine (TEA) and DEA as SDA, the crystallinity of synthesized SAPO-34 was lower than that obtained by DEA alone, and the crystallinity of SAPO-34 decreased with the increase in the amount of TEA in the mixed SDA (Li et al., 2005). However, the specific surface area and pore volume of SAPO-34 zeolite synthesized with DEA as SDA alone are lower than those synthesized with TEA as SDA alone. Figure 3 shows the XRD patterns of SAPO-34 zeolite synthesized with DEA (SAPO-34-M2), morpholine (SAPO-34-M6), and TEAOH (SAPO-34-M7) as SDA. It can be seen that when DEA was used as SDA, the crystallinity of the product is the highest. In contrast, the crystallinity of the product decreases when morpholine (MOR) is used as SDA, and when TEAOH is used as SDA, the heterophase of SAPO-5 zeolite appears in the product. Therefore, in this paper, DEA is mainly used as the SDA for the synthesis of SAPO-34 zeolite.

##### Effect of Crystallization Conditions

**FIGURE 4:**
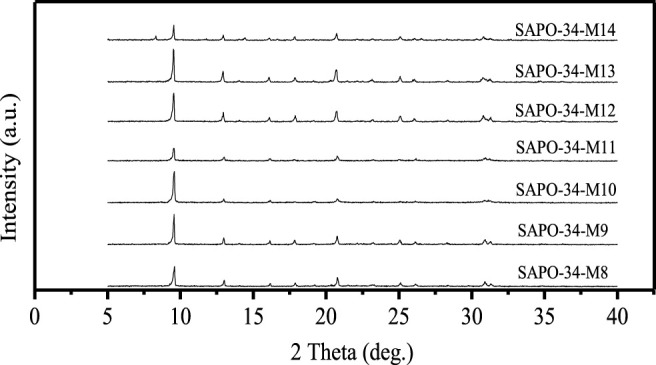
The XRD spectra of SAPO-34 samples synthesized at different crystallization temperatures and times.

The temperature and time of crystallization are important factors affecting the synthesis of all zeolites, because there are obvious differences between the skeletons of different zeolites, and the guiding mechanism of different SDAs is also different. With the increase in crystallization temperature, the induction period in the synthesis of zeolite will be shortened, and the formation and growth of crystals will be accelerated, but excessive crystallization temperature also provides conditions for the formation of the impure phase of zeolite. Figure 4 shows the XRD spectra of samples obtained with silica sol and pseudo boehmite as raw materials and a material ratio of 1.0 Al_2_O_3_:1.0 P_2_O_5_:0.6 SiO_2_:2.0 DEA:50 H_2_O at different crystallization temperatures and times: SAPO-34-M8: 150°C–24 h + 200°C–12 h; SAPO-34-M9: 150°C–24 h + 200°C–12 h; SAPO-34-M10: 170°C–24 h + 200°C–24 h; SAPO-34-M11: 180°C–72 h; SAPO-34-M12: 200°C–24 h; SAPO-34-M13: 200°C–48 h; SAPO-34-M14: 200°C–72 h. It can be seen that when crystallization was conducted in two stages (SAPO-34-M8, SAPO-34-M9, and SAPO-34-M10), the170°C of temperature of pre-crystallization (SAPO-34-M10) was better than 150°C (SAPO-34-M8, SAPO-34-M9), and the24 h of crystallization time for the second stage of crystallization is better (SAPO-34-M9). Moreover, when one-step crystallization is used, the crystallinity of obtained SAPO-34 is very low when the crystallization temperature is 180°C (SAPO-34-M11), so the crystallization temperature was raised to 200°C. The results showed that 48 h of crystallization time (SAPO-34-M13) brought about higher crystallinity than 24 h (SAPO-34-M12), but heterophase of SAPO-5 appeared in the product when the crystallization time is extended to 72 h (SAPO-34-M14). In conclusion, the better crystallization condition is 200°C–48 h (SAPO-34-M13).

**FIGURE 5:**
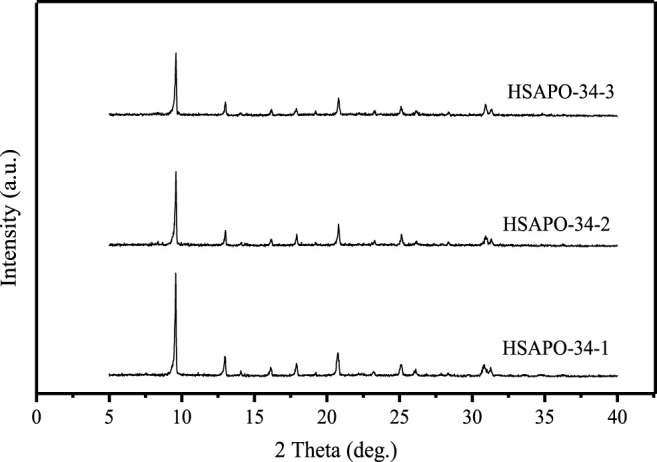
The XRD spectra of SAPO-34 samples synthesized with different ratios of materials.

Given all of those above, the conditions for synthesizing SAPO-34 zeolite with a mesoporous structure are as follows: silica sol and pseudo boehmite are used as inorganic silicon and aluminum sources, phosphoric acid is used as phosphorus source, DEA is used as microporous template, and organosilane DM-3010 is used as mesoporous SDA. The XRD spectra of the obtained HSAPO-34-1 zeolite molecular sieve is shown in Figure 5. It can be seen that the sample HSAPO-34-1 has higher crystallinity compared with the other two samples. It is proven that the addition of mesoporous SDA, namely, DM-3010, does not affect the crystallinity of the product (generally, the addition of mesoporous SDA will affect the formation of the zeolite crystal framework and decrease the crystallinity of the obtained sample, even to an amorphous product). The optimal initial raw material ratios are 1.0 Al_2_O_3_:0.6 SiO_2_:2.0 P_2_O_5_:3.1 DEA:90 H_2_O for HSAPO-34-1, 1.0 Al_2_O_3_:1.0 SiO_2_:1.5 P_2_O_5_:2.6 DEA:100 H_2_O for HSAPO-34-2, and 1.0 Al_2_O_3_:0.6 SiO_2_:1.1 P_2_O_5_:2.2 DEA:65 H_2_O for HSAPO-34-3. The volume ratio of DM-3010 to silica sol was 1:2 for synthesis of all hierarchical porous SAPO-34 zeolites.

#### N_2_ Adsorption–Desorption

The N_2_ adsorption–desorption results of HSAPO-34-1, HSAPO-34-2, and HSAPO-34-3 indicate that the existence of a mesoporous structure of HSAPO-34 zeolite and the pore size of HSAPO-34 is mainly concentrated at 4 nm, which provides favorable conditions for the catalytic conversion of a bulky organic molecule, such as 4,6-DMDBT, in the pore channel of zeolite. The texture properties of HSAPO-34-1, HSAPO-34-2, and HSAPO-34-3 are listed in Table 1. Compared with HSAPO-34-1, the BET-specific surface area and external surface area of both HSAPO-34-2 and HSAPO-34-3 are almost the same as that of HSAPO-34-1, but the micropore volume and mesopore volume decrease in varying degrees, which may result from the DEA content reduction in raw materials of HSAPO-34-2 and HSAPO-34-3, leading to the decrease in crystallinity and to the decrement of micropore volume of the sample. Besides that, the reduction of silicon content in the raw material reduces the silicon content in zeolite skeleton, which further reduces the interaction of zeolite skeleton and organosilane DM-3010, resulting in the decrement of mesoporous pore volume.

**TABLE 1:** The texture properties of HSAPO-34-1, HSAPO-34-2, and HSAPO-34-3.

Samples	*S*_BET_ ^a^ (m^2^/g)	*S*_ext_ ^b^ (m^2^/g)	*V*_micro_ ^c^ (cm^3^/g)	*V*_meso_ ^d^ (cm^3^/g)
HSAPO-34–1	754	133	0.25	0.23
HSAPO-34–2	758	148	0.20	0.20
HSAPO-34–3	580	165	0.17	0.22

a BET, specific surface area.

b External surface area (calculated from a pore diameter greater than 2 nm).

c Microporous volume (calculated from a pore diameter smaller than 2 nm).

d Mesoporous volume (calculated from a pore diameter greater than 2 nm).

#### Scanning Electron Microscope

**FIGURE 6:**
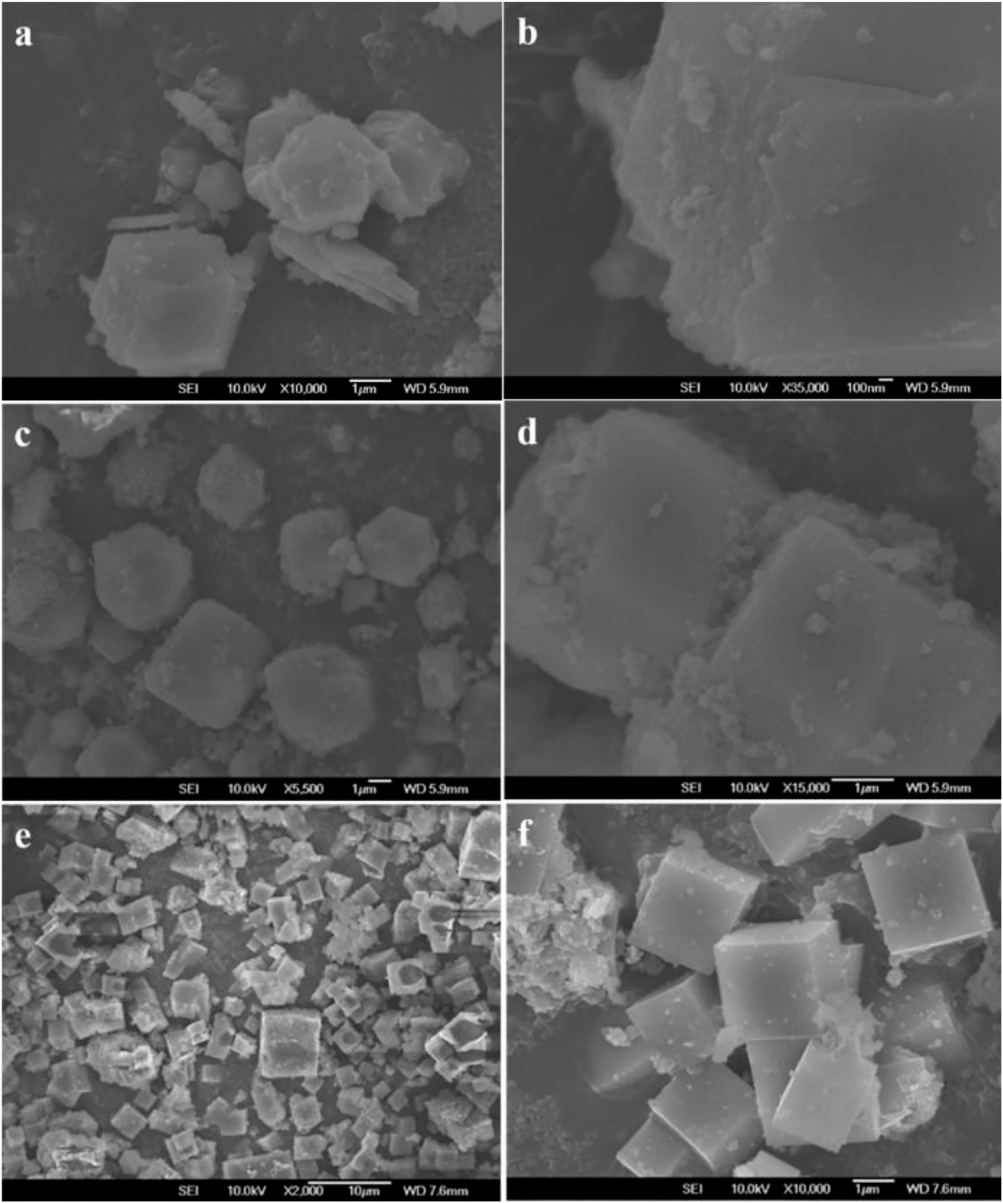
SEM images of HSAPO-34-1 **(A,B)**, HSAPO-34-2 **(C,D)**, and HSAPO-34-3 **(E,F)**.

Figure 6 shows the SEM images of HSAPO-34-1, HSAPO-34-2, and HSAPO-34-3. It can be seen that the size of the three samples is uniform, about 2–3 μm. Along with the increase in the mesoporous volume of the samples in the order of HSAPO-34-3, HSAPO-34-2, and HSAPO-34-1, the surface smoothness of the three samples decreases in turn, and the regular cube shape of the samples gradually becomes blurred, which is the result of the increase of mesoporous volume in zeolite skeleton.

#### Transmission Electron Microscope

**FIGURE 7:**
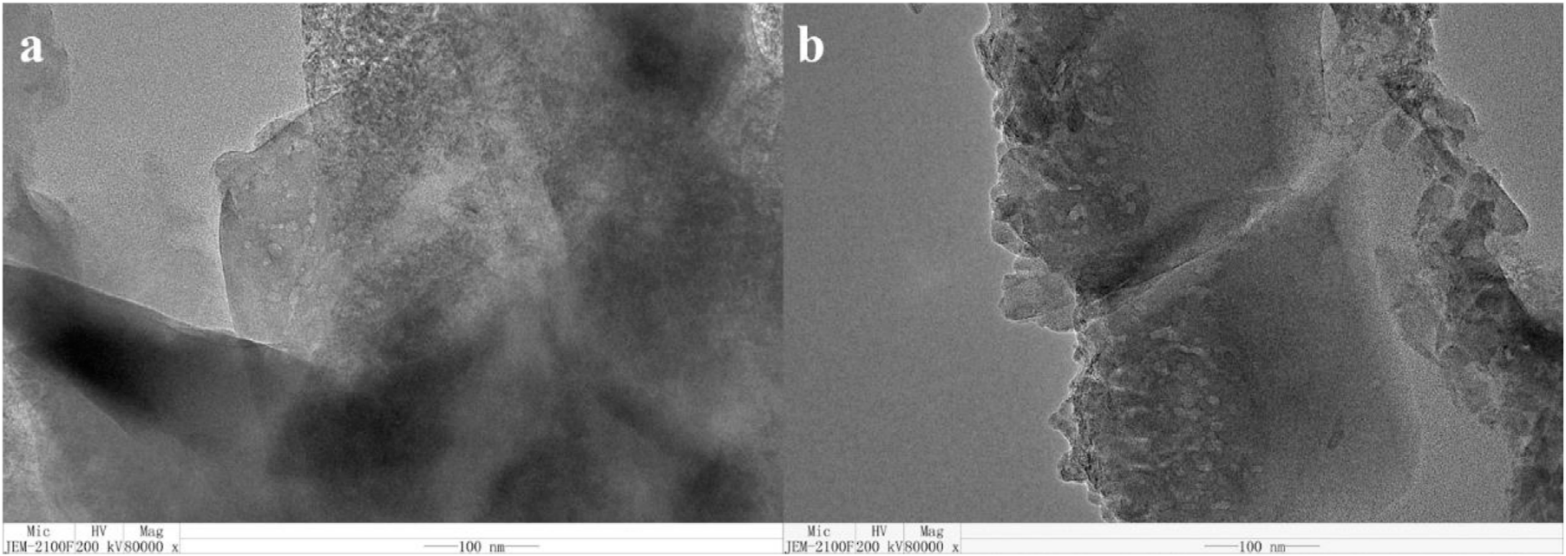
TEM image of HSAPO-34-1.

Figure 7 shows the TEM images of HSAPO-34-1. We can see many white small bright spots, that is, the mesoporous channel in HSAPO-34-1 with a size of about 5 nm, which is consistent with the N_2_ adsorption–desorption results about the pore diameter (4 nm). The TEM images give direct evidence for the existence of a mesoporous structure in HSAPO-34-1.

### Catalytic Performance of Hydrodesulfurization

The aim of the hydrodesulfurization process is to catalyze the sulfur-containing compounds in fuel oil at a certain temperature and pressure to convert them into corresponding hydrocarbons and H_2_S, so as to reduce the sulfur content in raw materials. The catalyst is the key factor for the efficiency of hydrodesulfurization. At present, Al_2_O_3_-supported metals Co and Mo are widely used in the industry to catalyze the conversion of organic sulfides in fuel oil. However, the surface Al_2_O_3_ suffers from the reduction of catalytic activity and instability, and it is difficult to treat macromolecular organic sulfides, such as dibenzothiophene (DBT) and 4,6-dimethyldibenzothiophene (4,6-DMDBT), to deeply affect hydrogenation. Therefore, it is very important to find a more effective carrier to overcome these weaknesses. In this work, a decahydronaphthalene solution of 4,6-DMDBT was used as simulated fuel oil, HSAPO-34-1 was used as carrier, and quantitative metal Co and Mo were loaded to characterize the hydrodesulfurization performance of HSAPO-34-1 at 5.0 MPa and 290°C and compared with the hydrodesulfurization performance of Al_2_O_3_ under the same conditions.

The hydrodesulfurization experiment of 4,6-DMDBT was carried out on a fixed bed. The inner diameter of the reactor was 6.5 nm and the length was 50 cm. After loading the prepared catalyst with a particle size of 0.245–0.350 mm into the reactor, *in situ* reduction with 10% H_2_–90% N_2_ mixture was conducted on a fixed bed. The temperature of the reactor was raised from room temperature to 300°C at a speed of 2°C/min and maintained at 300°C for 2 h, and the gas flow speed is 50 ml/min. Then, the reactor temperature and pressure were adjusted to 290°C and 5.0 MPa, and the inlet flow of reaction liquid and the flow rate of H_2_ were 5 ml/h and 60 ml/h, respectively. The reaction products took samples every 2 h and were detected using an Agilent 7890A GC with FID detector.

**FIGURE 8:**
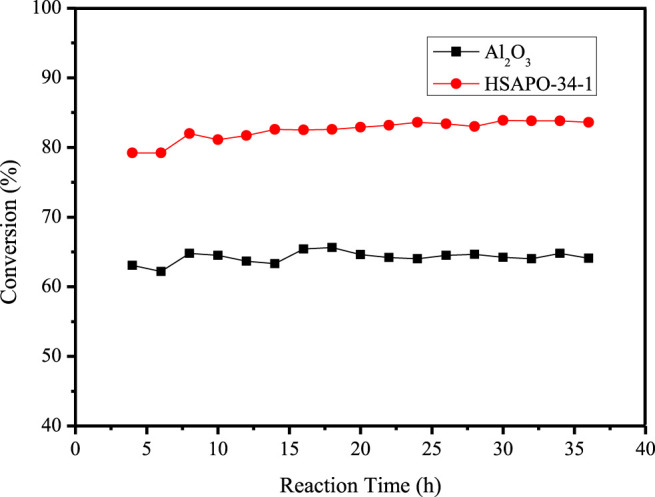
The hydrodesulfurization performance of supported HSAPO-34-1 and Al_2_O_3_.

Figure 8 shows the hydrodesulfurization performance of 4,6-DMDBT with HSAPO-34-1 and Al_2_O_3_ as carriers, respectively. It can be seen that the hydrodesulfurization conversion of 4,6-DMDBT with Al_2_O_3_ is about 64%, while the conversion with HSAPO-34-1 is about 83%. It is nearly 20 percentage points higher, which may be due to the medium-strength acid sites generated by the entry of silicon atoms into the skeleton of hierarchical porous HSAPO-34-1. Moreover, hierarchical porous HSAPO-34-1 not only provides larger mesoporous pore volume and pore diameter, but also has the skeleton structure of microporous zeolite, which can provide more active sites.

## Conclusion

In this work, the effects of different raw materials, material ratios, SDAs, and crystallization conditions on the synthesis of SAPO-34 zeolite were studied. On this basis, SAPO-34 zeolite with a hierarchical porous structure was characterized by XRD, N_2_ adsorption–desorption, SEM, and TEM. The obtained results prove that the synthesized SAPO-34 zeolite had good crystallinity and micro-mesoporous properties. Subsequently, the synthesized HSAPO-34 zeolite with a hierarchical porous structure was used as the carrier for catalytic hydrodesulfurization of bulky molecule organic sulfide 4,6-DMDBT, and also showed excellent performance. However, some aspects still need to be improved, such as whether SAPO-34 with a hierarchical porous structure can be synthesized with much cheaper mesoporous SDA. Additionally, the catalytic hydrodesulfurization of bulky molecule organic sulfide and kinetics of hydrodesulfurization with HSAPO-34 supported by different types and amounts of metals still need to be studied in the future.

## Data Availability

The original contributions presented in the study are included in the article/Supplementary Material. Further inquiries can be directed to the corresponding authors.
